# Genome sequences of fungi isolated from retail ground beef

**DOI:** 10.1128/mra.00732-26

**Published:** 2026-09-09

**Authors:** Chloe Kane, Ifigenia Geornaras, Peipei Zhang

**Affiliations:** 1 Department of Animal Sciences, Colorado State University 3447, Fort Collins, Colorado, USA; University of California Riverside, Riverside, California, USA

**Keywords:** fungi, ground beef, whole-genome sequencing, spoilage

## Abstract

Studies on fungi in meat products are very limited. In our previous study, we recovered 10 fungal strains from aerobically packaged ground beef collected from retail stores. We conducted whole-genome sequencing on these strains. This genomic information may help to inform more effective interventions to extend meat shelf life.

## ANNOUNCEMENT

Refrigerated meat spoilage is primarily driven by bacterial activity, though fungi can also contribute under certain conditions (1, 2). Numerous studies have investigated the bacteria contributing to meat spoilage; however, studies on fungi remain limited (2). In one of our previous studies (3) investigating spoilage-relevant bacterial communities in ground beef, we recovered 270 isolates from aerobically packaged ground beef collected from 6 retail stores (A–F) in Fort Collins, Colorado. Among these, 18 isolates were likely not bacteria but potentially fungi, as a DNA extraction method (3) that is generally effective for bacteria yielded very low amounts of DNA (5–235 ng) for these isolates. The majority (17/18) of these isolates were from retail store E, while one isolate was from store B. A total of 10 isolates were selected for whole-genome sequencing, with 1 representative isolate selected for each unique randomly amplified polymorphic DNA-PCR pattern identified within each ground beef package (4). The generated genomic information may help to inform more effective strategies to improve meat shelf life.

The 10 strains selected for sequencing were individually grown in tryptic soy broth (Difco, BD) at 25°C until reaching the stationary growth phase, which occurred after two (strains E113, E121, E122, and E212), three (E221), five (B615, E223, and E225), or six (E111 and E114) days. For DNA isolation, the DNeasy Blood & Tissue Kit (Qiagen) was used by mainly following the manufacturer’s protocol for gram-positive bacteria with an additional bead-beating step. Briefly, the pellet obtained from 1.8 mL of each culture was resuspended in 360 µL of enzymatic lysis buffer, transferred to a PowerBead Tube containing 0.5 mm glass beads (Qiagen), and vortexed at 3,000 rpm for 10 min. Following bead beating, 25 µL of Proteinase K (Qiagen) and 200 µL of Buffer AL (Qiagen) were added to each tube, and the mixture was incubated at 37°C for 1 h. The tubes were then centrifuged at 15,000 × *g* for 2 min. A 400 µL aliquot of the lysate was transferred to a new 2 mL microcentrifuge tube and processed starting from step 6 of the protocol mentioned above.

DNA library construction, sequencing, and genome assembly were conducted as previously described (3). Briefly, the genomes were assembled using SPAdes (v4.2.0) with the length of kmers set at 21, 33, 55, 77, and 99 (5). Contigs shorter than 500 bases in each genome were removed using a Python script (6). Mitochondrial sequences in the genomes were detected by the National Center for Biotechnology Information (NCBI) team using FCS (v0.5.5) (7) and manually removed from the assembled genomes. The completeness of each genome was assessed using BUSCO (v6.0.0) (8) in genome mode with “fungi_odb12” lineage data set and other settings at default. To determine the species identity of each genome, all fungal reference genomes available as of 24 March 2026 (*n* = 5,352) were downloaded using the NCBI data sets command-line tools (v18.21.0) (9) with default settings. Average nucleotide identity (ANI) between the 10 sequenced genomes and the reference genomes was calculated using FastANI (v1.34) (10) with default settings. A threshold of 95% was used for species delineation. The 10 strains were identified as *Tausonia pullulans* (B615), *Candida mesenterica* (E111 and E114), *Candida* sp. (E113, E121, E122, E212, E223, and E225), and *Yarrowia galli* (E221). The characteristics of sequencing data and assemblies are listed in Table 1.

**TABLE 1:** Summary of genome sequencing conducted for 10 fungal strains from aerobically packaged ground beef

Strain	Species	Best matched reference genome			G + C (%)	Assembly size (bp)	Number of sequencing reads	Genome coverage	No. of contigs	N50 (bp)	Genome completeness (%)	Accession number/name	
		Accession	Species	ANI with the sequenced genome (%)								SRA	Genome
B615	*Tausonia pullulans*	GCA_951802195.1	*Tausonia pullulans*	98.70	58.4	22,634,024	11,491,340	76.2	932	56,243	89.4	SRR37849020	ASM5821873v1
E111	*[Candida] mesenterica*	GCA_030559045.1	*[Candida] mesenterica*	99.92	44.2	14,262,809	9,584,848	100.8	59	1,336,623	81.7	SRR37849019	ASM5821859v1
E113	*[Candida]* sp.	GCA_977014415.1	*[Candida] sake*	93.25	38.8	14,149,786	11,184,478	118.6	54	1,308,224	83.2	SRR37849018	ASM5821855v1
E114	*[Candida] mesenterica*	GCA_030559045.1	*[Candida] mesenterica*	99.93	44.2	14,235,776	11,859,188	125.0	55	1,044,509	81.6	SRR37849017	ASM5821857v1
E121	*[Candida]* sp.	GCA_977014415.1	*[Candida] sake*	93.25	38.8	14,133,208	9,205,630	97.7	55	1,308,193	83.2	SRR37849016	ASM5821853v1
E122	*[Candida]* sp.	GCA_977014415.1	*[Candida] sake*	93.31	38.8	14,111,272	12,718,798	135.2	338	80,067	83.2	SRR37849015	ASM5821849v1
E212	*[Candida]* sp.	GCA_977014415.1	*[Candida] sake*	93.26	38.8	14,134,785	12,713,056	134.9	58	1,308,131	83.1	SRR37849014	ASM5821850v1
E221	*Yarrowia galli*	GCA_900519055.1	*Yarrowia galli*	99.95	49.3	22,719,204	11,488,012	75.8	170	365,377	89.4	SRR37849013	ASM5821846v1
E223	*[Candida]* sp.	GCA_030557765.1	*[Candida] saitoana*	86.11	34.4	13,716,089	12,937,434	141.5	136	502,489	80.8	SRR37849012	ASM5821845v1
E225	*[Candida]* sp.	GCA_030557765.1	*[Candida] saitoana*	86.18	34.4	13,695,500	12,048,048	132.0	434	72,228	80.8	SRR37849011	ASM5821843v1

## Data Availability

The raw sequencing data and the genome sequences have been deposited in GenBank (BioProject PRJNA1444087). The accession numbers for Sequence Read Archive (SRA) and genome assemblies are listed in Table 1.
